# Mechanisms of Enhancer-Promoter Interactions in Higher Eukaryotes

**DOI:** 10.3390/ijms22020671

**Published:** 2021-01-12

**Authors:** Olga Kyrchanova, Pavel Georgiev

**Affiliations:** Department of the Control of Genetic Processes, Institute of Gene Biology Russian Academy of Sciences, 34/5 Vavilov St., 119334 Moscow, Russia

**Keywords:** C2H2 proteins, CTCF, LDB1, chromatin insulator, long-distance interactions

## Abstract

In higher eukaryotes, enhancers determine the activation of developmental gene transcription in specific cell types and stages of embryogenesis. Enhancers transform the signals produced by various transcription factors within a given cell, activating the transcription of the targeted genes. Often, developmental genes can be associated with dozens of enhancers, some of which are located at large distances from the promoters that they regulate. Currently, the mechanisms underlying specific distance interactions between enhancers and promoters remain poorly understood. This review briefly describes the properties of enhancers and discusses the mechanisms of distance interactions and potential proteins involved in this process.

## 1. Introduction 

In higher eukaryotes, the regulation of gene expression is complicated as a consequence of cell differentiation during embryonic development [1,2,3]. Cell specialization is determined by differences in transcription factor (TF) repertoires, and the genes responsible for cell differentiation and organismal development are typically regulated by multiple independent enhancers, each of which stimulates a promoter in a limited population of cells during a specific time interval.

Enhancers were first described nearly 40 years ago when 72-bp tandem sequences from the SV40 virus were found to enhance gene expression when placed at large distances from the promoter and in any orientation relative to the regulated gene [4,5]. Two years later, the first cellular enhancer was identified [6]. Currently, the human genome is predicted to encode 300,000 enhancers [7].

Enhancers are regions of DNA, typically 100 to 1000 bp in size, that contain TF-binding sites that stimulate the initiation and elongation of transcription from promoters [1,8,9,10]. In most housekeeping genes, enhancers are located in close proximity to promoters, and distinguishing the borders between these regulatory elements can be difficult. In contrast, developmental genes typically feature complex regulatory regions that can consist of dozens of enhancers located at variable distances from the regulated promoter.

During transcriptional activation, enhancers are usually located in close proximity to gene promoters [8,11]. Here, we provide a brief overview of the currently available information in mammals and Drosophila regarding enhancers and describe in more detail the known architectural proteins that have been shown to support distance interactions between regulatory elements.

## 2. Enhancer Properties and Functions 

Enhancers consist of combinations of short, degenerate sites, 6–12 bp in length, that are recognized by DNA-binding TFs, which determine enhancer activity [1,9]. The combination of DNA-binding TFs on a given enhancer creates a platform that attracts co-activators and co-repressors that determine the enhancer activity in each specific group of cells. The ability of an enhancer to stimulate transcription depends on the combination of TF sites that positively or negatively affect enhancer activity and the relative concentrations of enhancer-binding TFs within the nuclei of a given group of cells. 

Recently, super-enhancers have been identified, representing a special class of regulatory elements, characterized by large sizes, sometimes reaching tens of thousands of bp, with a high degree of TF and co-activator enrichment [12,13]. Super-enhancers are often located adjacent to genes known to be critical for cell differentiation [14]. A more detailed study of super-enhancers has shown that they often consist of separate domains that can either function together to enhance the overall activity of each domain or play independent roles during the simultaneous activation of a large number of promoters [15,16,17].

**Figure 1:**
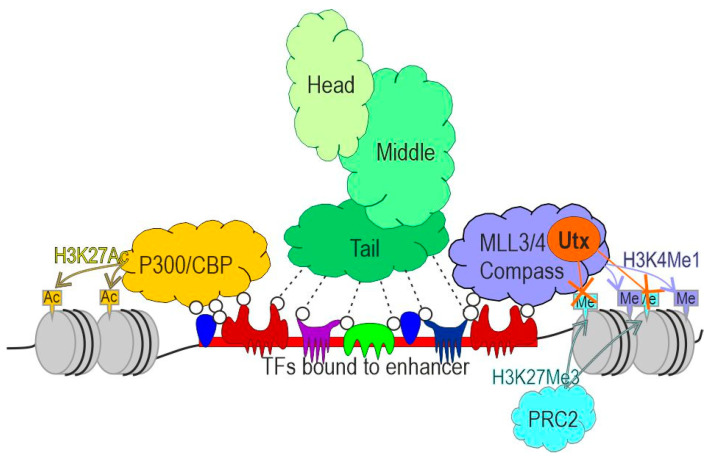
Schematic representation of the transcriptional complexes involved in enhancer activity. Various transcription factors (TFs) bind to enhancer sites and recruit complexes involved in transcription stimulation. p300/CBP possesses acetyltransferase activity and is responsible for H3K27 acetylation. The Mll3/4 complexes induce H3K4 monomethylation and recruit the UTX demethylase, which can remove the H3K27me3 generated by the PRC2 complex and associated with repressed chromatin. p300/CBP, Mll3/4, and UTX are thought to regulate transcription and enhancer activity through the modification of currently unknown components of transcriptional complexes on promoters. The subunits of the mediator complex form three main modules: head, middle, and tail. The mediator complex is recruited to the enhancer via multiple interactions between subunits of the tail module and the intrinsically disordered regions of TFs.

During the activation of transcription (Figure 1), enhancers recruit several key complexes. The p300/CBP and Mll3/Mll4/COMPASS complexes have acetyltransferase and methyltransferase activities, respectively [18]. The proteins Mll3 and Mll4 both contain a C-terminal SET (suppressor of variegation, enhancer of zeste, trithorax) domain, which is responsible for the monomethylation of lysine 4 of histone H3 (H3K4me1) [19,20]. The complexes formed by Mll3 and Mll4 have partially overlapping and insufficiently studied functions in the regulation of enhancer activity. Mll3 and Mll4 are also known to be involved in the recruitment of the p300/CBP co-activator, which is responsible for the acetylation of histone H3 at lysine 27 (H3K27ac) [21]. H3K27ac and H3K4me1 histone marks are distinctive features of active enhancers and are used to identify enhancers in genomes [22,23,24,25,26]. 

The H3K27ac and H3K4me1 modifications of histone H3 are thought to reduce the stability of nucleosomes [27], resulting in the formation of open chromatin and the stabilization of TF binding to enhancers. The Mll3/4 and p300/CBP complexes antagonize two Polycomb gene (PcG) complexes, Polycomb-repressive complexes 1 and 2 (PRC1 and PRC2, respectively), which are involved in the repression of enhancers and promoters [28]. PRC1 and PRC2 have ubiquitin transferase and methyltransferase activities, respectively, resulting in transcriptional repression [29]. The best-known activity of the PRC2 complex is the trimethylation of lysine 27 in histone H3 (H3K27me3), which is a characteristic marker of transcriptionally repressed chromatin regions. The Mll3 and Mll4 complexes are associated with UTX demethylase, which can remove the PRC2-deposited H3K27me3 [18], allowing the lysine 27 in H3 to be acetylated by p300/CBP, preventing the trimethylation (H3K27me3) by PRC2 and stabilizing the active chromatin in the enhancer region. Depending on their post- translational histone modifications [24,30,31], enhancers can be subdivided into poised (marked by H3K4me1 and H3K27me3) and active (marked by H3K4me1 and H3K27ac). During activation, poised enhancers lose the H3K27me3 mark and acquire H3K27ac in a cell type-specific manner [30]. In addition to the acetylation of H3K27, p300/CBP may play an important but poorly understood role in the acetylation of transcription factors involved in the pre-initiation complex formation [32,33]. 

A highly conserved mediator complex, consisting of 25 subunits in yeast and 30 subunits in humans, plays a primary role in the enhancer-mediated activation of transcription [34]. The subunits found in the mediator complex form three main modules: head, middle, and tail. The head and middle modules determine the primary functions of the complex during transcription activation, whereas the tail module is responsible for interaction with the TFs bound to enhancers and gene promoters [35,36,37,38,39]. Together with general transcription factors assembled on the promoter, the mediator complex facilitates the assembly of a pre-initiation complex and is involved in the recruitment of RNA polymerase II to promoters [40,41]. However, many of the processes that involve the mediator complex during the activation of transcription remain insufficiently studied [42]. 

## 3. Properties of Enhancer-Promoter Communication 

Most enhancers are located in close proximity to the promoters that they activate. However, even in the compact *Drosophila* genome, approximately 20% of enhancers are located at a distance of 50–100 kb from promoters associated with them, and often between them there can be other genes that have their own regulatory systems [43,44]. In mammals, the distances between enhancers and promoters are typically even larger [8]. Numerous experimental data support the idea that regulatory information for transcriptional control is transmitted through physical contact between enhancers and promoters [8]. 

Many distance interactions between various enhancers and promoters have been shown to be stable throughout *Drosophila* embryogenesis [44]. Similar results were obtained in mammalian cells, in which stable promoter-promoter and promoter-enhancer interactions were also identified [45,46]. Interactions between enhancers and promoters can form both before and during transcription activation [44,47,48]. Super-enhancers have been shown to simultaneously interact with a large number of promoters during cell differentiation [49]. Recently, the mediator complex was found not to be involved in the organization of distance interactions between enhancers and promoters [50], which is consistent with the finding that the mediator interacts only transiently with an enhancer [51]. 

Discontinuous, episodic bursts of transcriptional activity have been observed in a variety of systems and cell types [52]. The study of *β-globin* gene activation by a strong locus control region (LCR) enhancer in real time showed that enhancers activate transcription by increasing the frequency of intermittent transcriptional bursts [53]. Improving the stability of the interaction between the LCR and the *β-globin* gene promoter resulted in the increased frequency of transcription bursts. A similar study was performed on reporter genes in living transgenic *Drosophila* embryos [53], which revealed that the enhancer strength determines the burst frequency. Moreover, one enhancer can simultaneously, and in a coordinated manner, activate the promoters of two reporter genes but with different activation amplitudes [54].

**Figure 2:**
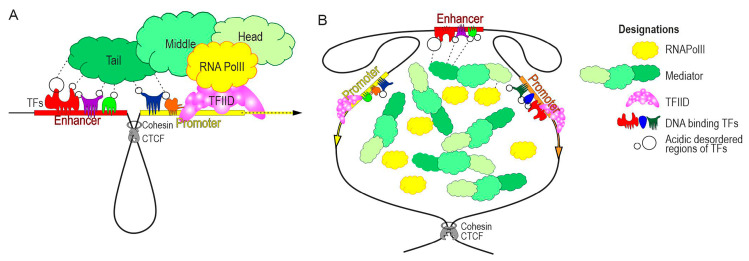
Models showing the remote activation of a promoter by an enhancer. (**A**) Classical model of enhancer-promoter communication. The interaction between an enhancer and a promoter can be stabilized by CCCTC-binding factor (CTCF), in cooperation with the cohesin complex and the dimerization of LOB domain-containing protein 1 (LDB1), which is recruited to chromatin through interactions with LIM proteins. The TFs bind to the enhancer and a promoter to form a platform for the transient recruitment of the mediator complex. The mediator complex transfers RNA polymerase II to the promoter transcription factor IID (TFIID) complex and accelerates further transcription initiation steps to induce a short transcriptional pulse (burst). The enhancer strength is directly correlated with the efficiency of mediator recruitment to chromatin. (**B**) Model of enhancer-promoter communication through the formation of hubs. Interactions between CTCF/cohesion sites form domains in which enhancers and promoters are located relatively close to each other. TF activation domains associated with enhancers usually contain internally disordered regions that can efficiently interact with subunits of the Mediator and RNA polymerase II complexes. As a result, the concentration of transcriptional complexes increases near enhancers, and promoters can more efficiently recruit these complexes to initiate transcription.

These results are consistent with two different but complementary models. According to the first model, the stable interaction between an enhancer and one or more promoters is established by specific architectural proteins. The close proximity between enhancers and promoters allows the TFs bound to these regulatory elements to form a platform to which the mediator complex and other transcriptional co-activators can transiently bind (Figure 2A). The unstructured acidic domains of TFs can interact with various subunits of the mediator complex, most of which are located in the tail module [55,56]. The efficiency of mediator recruitment is directly correlated with the number of TFs associated with enhancer-promoter sequences. During the transient binding with the enhancer-promoter complex, the mediator manages to induce only a short transcriptional pulse. The strength of transcription is determined by the burst frequency, which is directly dependent on the number of TFs that attract the mediator to the protein platform formed by the interaction between the enhancer and promoter. The second model (Figure 2B) is based on the emerging view that TFs dynamically bind and dissociate from their target sites, and occupancy is sustained by high local concentrations of TFs [57,58]. Many TFs and components of the basal transcription machinery can form condensates by their intrinsically disordered domains. TF condensates localized at enhancers recruit RNA polymerase II and Mediators to form an activation hub at target promoters [8,52]. As a result, enhancers need not directly interact with their target promoters, but merely come into relatively close proximity to them. The aggregation of multiple transcriptional complexes and RNA polymerase II could serve to bridge enhancers to their target promoters over such distances. This model explains well how super-enhancers can simultaneously activate a large number of promoters. Taking into account the many investigated interactions between enhancers and promoters, it can be assumed that the models as a whole complement each other.

## 4. Mechanisms of Distance Interaction between Enhancers and Promoters in Mammalian Genomes 

At a scale of tens to hundreds of kilobases, chromosomes fold into topologically associating domains (TADs) with preferential intradomain interactions compared to interdomain interactions with the neighboring *cis* chromatin domains [56]. The presence of these domains has been described in all well studied higher eukaryotes, indicating that they may represent a conserved feature of genome organization. TADs are architectural chromatin units that define regulatory landscapes, and genes tend to be coregulated during cell differentiation when they are located within the same TAD [59,60,61]. TAD boundaries are defined based on preferred interactions, and no known physical barriers restrict trans-interactions between regulatory elements located in neighboring TADs [59,61,62,63]. However, the TAD architecture can facilitate interactions between regulatory elements located within the TAD by reducing the physical distance between these elements. 

**Figure 3:**
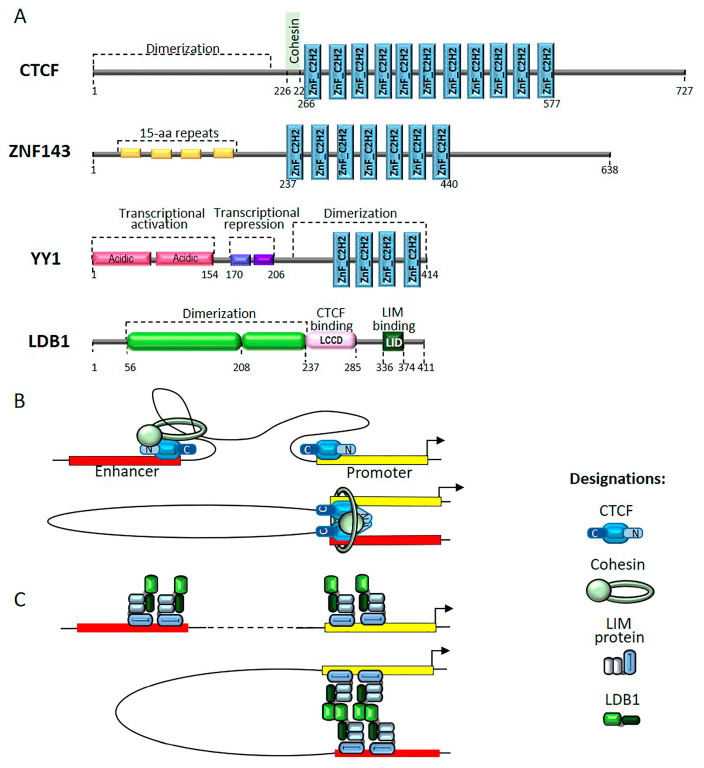
(**A**) Schematic representation of mammalian proteins involved in the formation of chromosome architecture. LDB1 contains LDB/Chip conserved domain (LCCD) that interacts with several DNA binding proteins including CTCF. (**B**) CTCF/cohesin loop formation model. (**C**) Model of LDB1-mediated enhancer-promoter interaction.

In mammals, most TAD boundaries contain binding sites for CCCTC-binding factor (CTCF) [64]. A characteristic feature of CTCF (Figure 3A) is the presence of a cluster, consisting of 11 C2H2-type zinc finger domains [65]. Five C2H2 domains in CTCF specifically bind to an extended DNA motif that is conserved in most animals [66]. The N-termini of CTCF homologs from representative bilaterian species feature unstructured domains that are capable of homodimerization [67]. A motif that interacts with the cohesin SA2-SCC1 sub-complex was identified between the N-terminal homodimerization domain and the C2H2 cluster (Figure 3A) [68].

Together with the cohesin complex, CTCF defines the boundaries of most TADs [11,61,69]. The CTCF/cohesin complex is also involved in organizing interactions between enhancers and promoters within TADs [70,71]. CTCF inactivation resulted in the re-localization of cohesin complexes from the CTCF binding sites to the promoters of active genes, which was accompanied by the partial disruption of the TADs [72]. Mutations in CTCF that disrupt the interaction with the cohesin complex also result in the loss of some distance interactions and a decrease in the efficiency of TAD formation [68,73]. 

Only a small fraction of CTCF sites located in the opposite convergent orientation are involved in TAD organization [74,75]. To explain the preferable formation of chromatin loops between CTCF sites located in a convergent orientation, a model was proposed in which the cohesin complex binds to the chromosome and initiates the extrusion of DNA, resulting in the formation of a chromatin loop (Figure 3B). CTCF can block the progression of the cohesin complex when the interaction between its N-terminal domain and the SA2-SCC1 sub-complex [68] is oriented correctly relative to the moving cohesin complex (Figure 3B). The dimerization between the N-terminal domains of the CTCF protein is thought to stabilize the formation of chromatin loops [67]. 

Regulation of the *Sonic hedgehog* (*Shh*) gene is a example for the role TAD organized by CTCF/cohesion complexes in increasing the probability of long-range enhancer-promoter interactions [8]. *Shh* is a pleiotropic developmental gene, that is regulated by multiple tissue specific enhancers in many organs such as the brain, lung and limbs [76]. The unique limb enhancer is located almost a 1 MB away from the *Shh* promoter within the intron of the constitutively expressed gene, *Lmbr1* [77]. Both genes are located in the same TAD formed by CTCF sites. When the TAD structure is altered by an inversion, the limb enhancer-*Shh* interaction is diminished [78]. However, *Shh* expression was restored by reducing the genomic distance separating the enhancer and the promoter within the inversion chromosome. Role of TAD boundaries was directly tested by deletion of the CTCF sites [79]. The CTCF/cohesin-mediated preformed topology of the *Shh* locus has been found to maximize gene expression in vivo, but enhancer-mediated activation also persists in TAD disruption. There are many examples demonstrating the role of TAD in facilitating correct and preventing incorrect interactions between enhancers and promoters [80,81,82,83]. However, inactivation of TADs throughout the genome has a relatively weak effect on global gene expression [72,84,85,86,87], suggesting that local interactions between enhancers and promoters plays a major role in the regulation of transcription.

There is evidence that CTCF is only one of many proteins involved in the organization of chromosome architecture. Inactivation of CTCF usually leads only to partial disruption of chromatin loops and recruiting of cohesin complexes [72,88]. During cell senescence, cohesion binds to chromatin independently from CTCF and form new chromatin loop domains associated with highly active genes [89]. To date, only a few proteins have been described that can potentially participate in the organization of chromosome architecture in mammals [11]. Two of these proteins, zinc finger protein 143 (ZNF143) and Yin Yang1 (YY1), bind DNA using C2H2 domains.

ZNF143 shares similarity with CTCF and the central region of ZNF143 contains a cluster that consists of seven C2H2 domains, three of which bind to a specific CCCAGCAG motif (Figure 3A) [90]. The N-terminal domain contains three 15-aa repeats with unknown functions, and the C-terminal domain is enriched in acidic amino acids, which is typical of transcription activators. ZNF143 is essential for embryonic development in mammals [91]. ZNF143 functions in the promoter region by participating in the formation of open chromatin regions and the recruitment of complexes that activate transcription [92]. Genome-wide studies have shown that ZNF143 can participate in the formation of chromatin loops between enhancers and promoters [93,94,95]. In human HEK293T cells, ZNF143 functions together with CTCF to form chromatin loops at several genomic sites [95]. However, in contrast to CTCF, no experimental evidence has suggested that ZNF143 participates in the localization of the cohesin complex on chromatin. Thus, how ZNF143 supports specific distance interactions remains unknown.

The mammalian YY1 protein (Figure 3A), which consists of only 414 amino acids, belongs to a multifunctional, evolutionarily conserved family of mammalian transcription factors and contains 4 C2H2 domains at the C-terminus [96]. *Drosophila* expresses two homologs of the YY1 protein, PHO and PHOL, which are involved in the recruitment of Polycomb proteins [97]. The N-terminal region of YY1 has been implicated in transcriptional activation, whereas the domain between 201–226 amino acids is involved in the recruitment of PcG proteins that are responsible for repression (Figure 3A) [98,99,100].

YY1 is predominantly associated with gene enhancers and promoters, which is consistent with a potential role in distance interactions [101,102]. The inactivation of YY1 results in a marked decrease in the number of distance interactions in vitro [101]. According to the proposed model, YY1 forms homodimers, which can bring the associated gene enhancers and promoters closer together. A cluster of C2H2 domains and an adjacent unstructured domain are responsible for the dimerization and oligomerization of YY1 [103,104]. Moreover, the dimerization and subsequent oligomerization of YY1 results in non-specific binding to DNA, especially with guanine quadruplexes (G4). Some experimental evidence has suggested that the dimerization of YY1 and the subsequent binding to G4 structures contribute to the YY1-mediated formation of long DNA loops [104]. YY1 may also participate in the organization of distance interactions through the regulation of proteins that are directly involved in the formation of chromatin loops. For example, YY1, together with Oct4, participates in the recruitment of the BAF remodeling complex to promoters and super-enhancers [105]. BAF can improve TF binding and stabilize the chromatin loops formed by CTCF/cohesin [102]. Therefore, YY1 appears likely to regulate enhancer activity and enhancer-promoter interactions through epigenetic mechanisms [106].

The role of the small protein LIM domain-binding factor 1 (LDB1) in the maintenance of distance interactions between enhancers and promoters has been studied in detail [107]. Unlike C2H2 proteins (Figure 3A), LDB1 binds to enhancers and promoters through the interaction between its C-terminal domain, named LIM interacting domain (LID), and DNA-binding TFs that belong to the LIM family [107]. Through interactions with various LIM partners, LDB1 plays roles in several regulatory processes during embryonic development and cell differentiation, including erythropoiesis. Initially, the N-terminal domain of LDB1 was shown to be involved in the organization of interactions between a strong enhancer (LCR) and the promoters of the *beta-globin* locus (Figure 3A) [108,109]. Structural analysis showed that the N-terminal domain of LDB1 contains both alpha helices and beta sheets, which form a stable homodimer [110,111]. TFs in the LIM family predominantly bind to gene enhancers and promoters, facilitating the recruitment of LDB1 to these regulatory elements. According to the model, specific interaction between the N-terminal domains of LDB1 molecules associated with enhancer and promoter elements can stabilize distance interactions between these regulatory elements (Figure 3C). Interestingly, LDB1 not only homodimerizes but can also interact with CTCF, which can promote the organization of contacts between enhancers and promoters associated with a large group of erythroid genes [86]. A small domain in LDB1, located near the N-terminal dimerization domain, interacts with an unidentified C2H2 domain in the CTCF protein. However, whether the interaction between the LDB1 domain and the C2H2 domain in CTCF is capable of forming a sufficiently stable and specific interaction between enhancers and promoters to regulate gene expression remains unknown. Recently, mutations in LDB1 that disrupt dimerization were shown to have no effect on the transcriptional activation of *beta-globin* genes [111], which suggested the existence of additional mechanisms to support the interaction between the LCR enhancer and *beta-globin* promoters. Therefore, LDB1 likely acts in cooperation with other unidentified proteins to support distance interactions.

## 5. Specific Activation of Olfactory Receptor Genes in Mammals Is Supported by Super-Long-Distance Interactions between Enhancers and Promoters 

**Figure 4:**
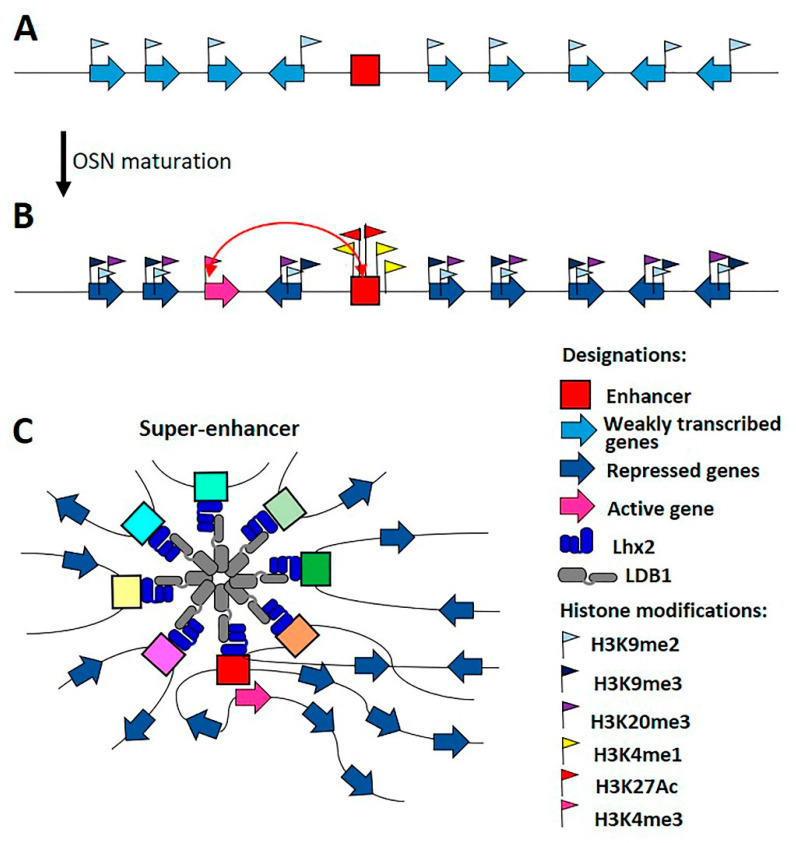
Model of the formation of highly specific transcription hub by OSN enhancers. (**A**) In progenitor cell, all *OR* genes are weakly co-expressed. (**B**) During OSN maturation, transcription of one *OR* gene is activated randomly, while all others remain completely suppressed. The *OR* genes are enriched in chromatin marks (H3K9me3, H3K20me3) associated with transcriptional repression. The transcriptionally active *OR* promoter, labeled H3K4me3, is associated with a hub formed by enhancers enriched in the Edf and Lhx2 proteins and nucleosomes marked by H3K4me1 and H3K27Ac. (**C**) The LDB1 protein recruited by Lhx2 is involved in the organization of hub formed by OSN enhancers located on different chromosomes. It seems likely that other unknown architectural proteins are involved in hub formation in cooperation with LDB1.

The most interesting models for understanding the mechanisms of distance interactions are proven examples of interactions between enhancers and promoters that are separated by megabase distances. In mammals, expression mechanisms associated with a large family of genes encoding olfactory receptors have been well-studied [112]. In the mouse genome, approximately 1100 genes encoding olfactory receptors (ORs) have been identified [113,114]. These genes are located in 40 clusters that are scattered throughout the mouse genome. Olfactory sensory neurons (OSNs) are derived from progenitor cells, in which all *OR* genes are very weakly co-expressed (Figure 4A). Constitutive heterochromatin is formed on inactive *OR* genes, which are enriched in H3K9me3 and H4K20me3 histone modifications [115]. During OSN maturation, the transcription of one *OR* gene is activated randomly, while all others remain completely repressed (Figure 4B) [116]. A negative feedback loop likely exists, in which the strong expression of one OR receptor results in the complete inactivation of all other *OR* genes [117,118,119]. However, the exact mechanism associated with this process remains poorly understood. 

Each cluster of *OR* genes has a nearby enhancer, which is involved in the selection of one gene that will be actively transcribed in a particular OSN [120,121]. A total of 14 specific enhancers have been identified, and the in vivo deletion of three of these enhancers has been shown to result in the complete inability to activate any of the *OR* genes encoded in the corresponding cluster [120,122,123,124]. A number of studies have shown that enhancers form a single cluster in the nucleus, which regulates the activation of a single selected *OR* gene (Figure 4C) [124], and all other genes form heterochromatin. Thus, active chromatin appears to be generated by the interactions between enhancers and the promoter of a single *OR* gene that is encoded in close proximity to clusters of *OR* genes to organize heterochromatin regions.

The mechanisms underlying the physical separation and stable balance between regions containing both active and repressed chromatin remain poorly understood. *OR* gene promoters and enhancers contain binding sites for Ebf and Lhx2, which are specific TFs expressed in neurons [20,121,124]. According to the model [112], the formation of a cluster of enhancers can increase the efficiency of Ebf and Lhx2 recruitment, resulting in the significant enrichment of transcription activators associated with the active *OR* promoter compared with the promoters associated with repressed *OR* genes (Monahan et al., 2017). A high concentration of activators can also prevent the spread of heterochromatin to active promoter associated with enhancers.

The most important aim is the identification of proteins that can support the specific super-long-distance interactions between enhancers that form a single cluster. CTCF and cohesin were not identified in the regulatory regions of *OR* genes. However, Lhx2 has been shown to recruit LDB1 to *OR* enhancers, and the inactivation of LDB1 has been shown to result in the partial disruption of *OR* enhancer colocalization in the nucleus [125]. These results suggest a potential role for LDB1 in the organization of super-long-distance interactions among *OR* enhancers. However, the most likely scenario is that several unknown architectural proteins are involved in the initiation and maintenance of distance interactions and that LDB1 facilitates their functions. 

## 6. Super-Long-Distance Interactions in the *Drosophila* Genome and the Role of Architectural Proteins during This Process

In the model organism *Drosophila*, there are no well-described interactions between enhancers and promoters at super-long distances. However, an efficient method for obtaining integrations into the *Drosophila* genome of single copies of constructs based on the *P*-transposon was created long ago [126]. This method allowed to demonstrate super-long-distance interactions between regulatory elements located at different genomic sites for the first time among higher eukaryotes. 

The first work on this topic [127] tested the effects of an insulator found in the *gypsy* retrotransposon on repression of a reporter gene induced by a Polycomb-dependent silencer [Polycomb response element (PRE)]. This study used a 660-bp PRE, found in the regulatory *bxd* region of the homeotic *Ubx* gene of the *bithorax* locus (*bxd* PRE). Typically, pairing two PRE-containing transgenes results in the increased repression of the reporter, which is associated with an increase in the recruitment efficiency of PcG complexes [29]. The combination of two transgenes that both contained PREs and the *gypsy* insulator resulted in the significant repression of reporter genes [127], despite these genes being separated by several megabases or even located on different chromosomes. The interaction between *gypsy* insulators can facilitate functional interaction between the enhancer and the promoter located in different transgenes inserted into the genome at distances reaching 1–2 megabases [128]. 

A similar study was performed with the insulators from the *bithorax* complex. The *Mcp* boundary separates the regulatory domains of the homeotic *abd-A* and *Abd-B* genes and consists of an insulator, which is flanked on both sides by Polycomb-dependent silencers [129]. Transgenes containing the *Mcp* were inserted in different regions of the third chromosome. The results indicated that combinations of transgenes located at a distance of several megabases cause an increase of marker gene repression that assume the physically interaction between them. In another work, the *bxd* PRE, in combination with the 210-bp core of the *Mcp* insulator, was able to support repression between two transgenes located at super-long-distances [130]. The co-repression of reporters and their colocalization in the nucleus were observed only in the presence of the *Mcp* insulators for both tested transgenes [130,131,132]. 

Super-long-distance interactions can be also be maintained by the *Fab-7* boundary, which separates the domains of the *Abd-B* regulatory region in the *bithorax* complex [133]. A 3.6-kb DNA fragment of the *Fab-7* boundary that included insulator and an adjacent PRE located on X-chromosome functionally interacted with endogenous *Fab-7* [133]. The pairing between the 1250-bp *Fab-7* insulators was also able to support super-long-distance interactions between transgenes in the *Drosophila* genome [130].

Two insulators, *Neighbor of Homie (Nhomie*) and *Homing* insulator at *eve* (*Homie*), were identified at the 16-kb boundaries of the regulatory region of the *even-skipped* (*eve*) gene [134,135,136]. *Nhomie* and *Homie* interact with each other and can also maintain super-long-distance interactions between the transgene and the endogenous *eve* locus, which allows endogenous enhancers to activate the reporter gene promoter in the transgene [135,136].

The interaction specificity between identical regulatory elements is also manifested in the “homing” phenomenon, in which the *P*-transposon containing either an insulator, or a promoter region, is inserted with high frequency into the genomic region where this regulatory element is located. For example, the *P*-transposon that contains the promoter region of *engrailed* or *linotte* was predominantly inserted (20–30%) into the corresponding genes [137,138]. Similar results were obtained upon integration into the genome of the *P*-transposon containing the *Fub* boundary, which organizes TADs that separate the regulatory regions of the *Ubx* and *abd-A* genes in the *bithorax* complex [139]. The “homing” effect occurs due to the interaction between architectural proteins, those are associated with two identical regions in an endogenous locus and *P-*transposon and directs the integration of the *P* transposon into the region of the corresponding gene.

In all of these examples, super-long-distance interactions are supported by a pair of identical regulatory elements that are unique to the genome. The *gypsy* insulator consists of 12 Su(Hw) binding sites [140,141,142]. In the genome of most Drosophila lines, the *gypsy* retrotransposon is found only in heterochromatin [143]. On the other hand, only a small fraction of several thousand Su(Hw) regions contain 2–3 binding motifs for this protein [144]. Thus, the *gypsy* insulator is unique due to a large number of Su(Hw) binding sites. According to ModEncode, the *homie* insulator contains binding sites for the *Drosophila* homolog of CTCF (dCTCF), Su(Hw), and GAF proteins [144]. The *Mcp* boundary contains binding sites for dCTCF and Pita [145]. The *Fub* boundary contains binding sites for CTCF, Su(Hw), and Pita [145,146,147]. Similar to dCTCF, Su(Hw) and Pita have clusters of C2H2 domains, some of which facilitate the specific binding of these proteins with DNA motifs [148,149,150]. Finally, the *Fab-7* boundary consists of three DNase 1 hypersensitivity sites (HS) [151,152,153]. HS2 has two Pita sites [145], whereas the central HS1 domain contain six GAF binding sites that overlap with long, degenerate binding sites for the newly identified late boundary complex (LBC) [154]. LBC is likely to be involved in the regulation of distance interactions between enhancers and promoters [154,155]. In all of the above examples, several proteins work together to organize active boundaries/insulators that are capable of supporting super-long-distance interactions. 

In transgenic model systems, the pairing between two copies of four or five binding sites of dCTCF, Su(Hw), or Pita was able to bring the yeast GAL4-dependent activator region and the reporter gene promoter in close proximity, resulting in the transcription of the reporter gene [156,157,158]. The dCTCF N-terminus contains an unstructured domain that can form tetrameric complexes [67,159], which can contribute to distance interactions. Similarly, Pita contains an N-terminal zinc-finger-associated domain (ZAD) capable to forming homodimers [158]. ZADs have also been identified in the N-termini of approximately one hundred *Drosophila* proteins containing clusters of C2H2 domains (ZAD-C2H2) [160,161]. An important feature of ZADs is their preferentially ability to homodimerize into an antiparallel dimer [161]. In addition to Pita, several other ZAD-C2H2 proteins, including ZIPIC (Zinc-finger protein interacting with CP190), Zw5 (Zeste-white 5), and ZAF1, have been shown to support distance interactions and form functional insulators [150,162,163,164,165]. Mutational analysis, *in vivo,* showed that the presence of ZADs determined the ability of these proteins to support distance interactions [158,165]. In transgenic lines, combinations of repeating binding sites for different ZAD-C2H2 proteins were unable to support the distance activation of the reporter gene by the GAL4 activator [156,158]. Thus, the homodimerization of ZADs is an important feature required to support specific distance interactions between ZAD-C2H2 proteins. 

The C2H2 proteins, including Pita, dCTCF, and Su(Hw), interact with BTB-containing proteins (bric-a-brac, tramtrack, and broad complex), such as Mod(mdg4) and CP190 [150,166,167,168]. The CP190 protein contains a classical N-terminal BTB domain that forms homodimers and is conserved among higher eukaryotes [169,170,171]. In contrast, the BTB domain of Mod(mdg4) belongs to an insect-specific group [172,173]. The BTB domains in this group of proteins can form both homo- and heteromultimeric complexes [171]. Previously, both CP190 and one of the Mod(mdg4) isoforms, named Mod(mdg4)-67.2, were thought to be capable of participating in the formation of distance interactions between regulatory elements and Su(Hw)-dependent insulators [174,175]. Both proteins are required for the functional activity of the *gypsy* insulator [168,176,177,178]. However, the CP190 and Mod(mdg4) can also interact with a large variety of DNA-binding proteins [150,159,166,167,168,179,180,181], which is inconsistent with their key role in the organization of specific distance interactions between *gypsy* insulators. Most likely, CP190 and Mod(mdg4) are involved in stabilizing pre-formed specific distance interactions. 

**Figure 5:**
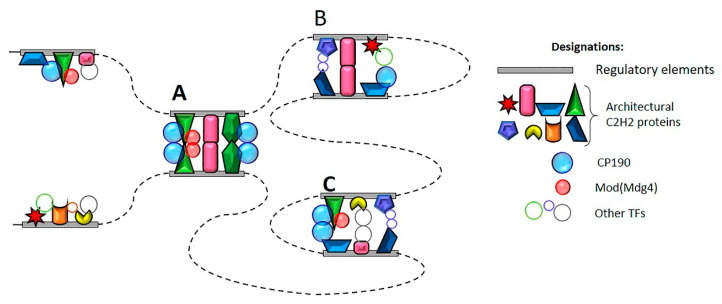
Model of interaction between regulatory elements in Drosophila. Architectural C2H2 proteins bind in different combinations to regulatory elements (promoters, enhancers, insulators). The CP190 and Mod(mdg4) and other similar proteins with homodimerization domains are recruited on the regulatory elements through interaction with C2H2 proteins. (**A**) Super-long-distance interactions are supported by multiple interactions between 3–6 C2H2 proteins associated with the same or structurally similar insulators. The interactions between proteins like Mod(mdg4) and CP190 can play a role in maintaining of stable interaction. (**B**) Specific distance interactions (5–20 kb) can be supported by regulatory elements that contain only partially similar combinations of C2H2 proteins. The auxiliary CP190 and Mod(mdg4) can play an important role in maintaining remote communications in such cases. (**C**) Local interactions between regulatory elements can be supported by proteins such as CP190 and Mod(mdg4), which can be recruited to completely different combinations of C2H2 proteins.

According available data the well described C2H2 proteins bind predominantly to promoter and insulator/boundary elements [148,149,150,158,165,181]. Based on the evidences presented above, we suggest the model [161] that all regulatory elements are formed by different combination of binding sites for different C2H2 proteins (Figure 5). The stability of the contact between regulatory elements depends on the presence of C2H2 proteins that can form homodimmers. Proteins like CP190 and Mod(mdg4) can support local interactions between regulatory elements and also could contribute for the long-distance interactions between them. 

## 7. Conclusions

It is now believed that the formation of chromatin architecture in mammals and Drosophila occurs using different mechanisms. This conclusion is based on the observation that the *Drosophila* CTCF protein is not key in the formation of TADs and does not have intense colocolization with the cohesin complex on chromatin [182,183,184]. Also, unlike mammals, the TADs boundaries in *Drosophila* are predominantly located in the regions of housekeeping gene clusters, which are actively transcribed.

However, it has recently been shown that dCTCF is located at the TAD boundaries in *Drosophila* nerve cells [185]. In *Drosophila*, the cohesin complex is predominantly located in the region of active promoters and enhancers, which is consistent with its potential role in the organization of TADs and the formation of interactions between enhancers and promoters [186,187]. Mutations in the *Nipped-B* gene that regulates the binding of the cohesin complex to chromatin affect the distance interactions between enhancer and promoter at the *cut* locus [188]. The mammalian LDB1 protein has a *Drosophila* homolog called Chip, which is also involved in maintaining distant enhancer—promoter interactions [189,190]. A direct interaction has been shown between the Chip and C2H2 domains of the Su(Hw) protein [191], which resembles the interaction described above between the LDB1 and C2H2 domains of the human CTCF protein [86]. Thus, the mechanisms of distance interactions may be much more conservative between mammals and *Drosophila* than it currently seems. Probably in the near future it will be possible to create a unified model of distance interactions in higher eukaryotes. 

There is more and more experimental evidence that in mammals other C2H2 proteins can participate in the formation of distance interactions and interact with CTCF in this process. The identification of binding sites for currently uncharacterized human and *Drosophila* C2H2 proteins will facilitate the assessment of the true contributions of this class of proteins to the organization of the chromosomal architecture. In addition, the use of gene editing methods, such as the CRISPR/Cas9, will make it possible to assess the role of each identified C2H2 protein in maintaining distance interactions using model regulatory systems. 

## Data Availability

Not applicable.

## Abbreviations

TF	Transcription factor
CBP	CREB-binding protein
Mll3/4	Mixed lineage leukemia 3/4
UTX	Demethylase
PcG	Polycomb group
PRC1/2	Polycomb repressive complexes 1/2
CTCF	CTC binding factor
ZNF143	Zinc finger protein 143
LDB1	LIM domain—binding factor 1
CP190	Centrosomal protein 190kD
Mod(mdg4)	Modifier of mdg4
CRISPR	Clustered regularly interspaced short palindromic repeats
